# Novel Nanoconjugate of Apamin and Ceftriaxone for Management of Diabetic Wounds

**DOI:** 10.3390/life12071096

**Published:** 2022-07-21

**Authors:** Abdullah A. Alamoudi, Awaad S. Alharbi, Ashraf B. Abdel-Naim, Shaimaa M. Badr-Eldin, Zuhier A. Awan, Solomon Z. Okbazghi, Osama A. A. Ahmed, Nabil A. Alhakamy, Usama A. Fahmy, Ahmed Esmat

**Affiliations:** 1 Department of Pharmaceutics, Faculty of Pharmacy, King Abdulaziz University, Jeddah 21589, Saudi Arabia; aalamoudi1@kau.edu.sa (A.A.A.); awaadalharbi@hotmail.com (A.S.A.); sbadr5@hotmail.com (S.M.B.-E.); oaahmed@kau.edu.sa (O.A.A.A.); nalhakamy@kau.edu.sa (N.A.A.); uahmedkauedu.sa@kau.edu.sa (U.A.F.); 2 Alrass General Hospital, Ministry of Health, Qassim Region, Ar Rass 58883, Saudi Arabia; 3 Department of Pharmacology and Toxicology, Faculty of Pharmacy, King Abdulaziz University, Jeddah 21589, Saudi Arabia; abnaim@yahoo.com; 4 Department of Pharmaceutics, Faculty of Pharmacy, Cairo University, Cairo 11562, Egypt; 5 Department of Clinical Biochemistry, Faculty of Medicine, King Abdulaziz University, Jeddah 21589, Saudi Arabia; zawan@kau.edu.sa; 6 Global Analytical and Pharmaceutical Development, Alexion Pharmaceuticals, New Haven, CT 06510, USA; solomon.z.okbazghi@gmail.com; 7 Center of Excellence for Drug Research and Pharmaceutical Industries, King Abdulaziz University, Jeddah 21589, Saudi Arabia; 8 Mohamed Saeed Tamer Chair for Pharmaceutical Industries, King Abdulaziz University, Jeddah 21589, Saudi Arabia; 9 Department of Pharmacology, Faculty of Medicine, King Abdulaziz University, Jeddah 21589, Saudi Arabia; 10 Department of Pharmacology and Toxicology, Faculty of Pharmacy, Ain Shams University, Cairo 11566, Egypt

**Keywords:** anti-microbial peptides, ceftriaxone, apamin, hydrogel, diabetic wounds, optimization, three-factor Box–Behnken design

## Abstract

Diabetic hyperglycemia delays wound healing, leading to serious consequences. Topical antibiotics can reduce the risk of a wound infection during healing; nevertheless, the microbial fight against antibiotics brings about public health challenges. Anti-microbial peptides (AMPs) belong to a novel class of drug that is used to prevent and treat systemic and topical infections. The aim of the current work was to achieve better wound healing in diabetic rats by conjugating the anti-microbial peptide “apamin” (APA) with the broad-spectrum antibiotic “ceftriaxone” (CTX) to form a nanocomplex. The CTX–APA nanoconjugate formulation was optimized using a Box–Behnken design. The optimized CTX–APA nanoconjugate formulation was evaluated for its size and zeta potential, and was then examined using transmission electron microscopy (TEM). The CTX–APA nanoconjugate was loaded onto a hydroxypropyl methylcellulose (2% *w*/*v*)-based hydrogel. It was observed that the application of the CTX–APA nanocomplex on the wounded skin of diabetic rats accelerated the regeneration of the epithelium, granulation tissue formation, epidermal proliferation, and keratinization. The nanocomplex was capable of significantly reducing the expression of tumor necrosis factor alpha (TNF-α) and interleukin 6 (IL-6), while increasing the expression of transforming growth factor beta-1 (TGF-β1) as well as the angiogenic markers: hypoxia-inducible factor 1-alpha (HIF-1α) and vascular endothelial growth factor (VEGF). Conclusively, the application of an ion-paired CTX–APA nanocomplex enhances wound healing in diabetic rats.

## 1. Introduction

Diabetes mellitus (DM) is a metabolic syndrome characterized by prolonged hyperglycemia. The development of DM is owing to either insufficient insulin production by the pancreas or the lack of a proper response to insulin by the body’s cells [1]. It is expected that diabetes will become one of the most prevalent illnesses in the Middle East and North Africa (MENA) because of obesity, rapid urbanization, a lack of exercise, and changes to how people live in the region [2]. It has been estimated by the International Diabetes Federation (IDF) that in 2019, 55 million adults between the ages of 20 and 79 suffered from diabetes in the MENA region. Further, the IDF estimates a rise in this figure to 108 million by 2045. Hyperglycemia affects about 11% of live births during pregnancy in the MENA region [3]. The World Health Organization (WHO) reports that Saudi Arabia is second in the list of countries where diabetes is most prevalent within the Middle East region, and seventh worldwide. The number of adults that have diabetes in Saudi Arabia is 4,275,200 [4]. 

Diabetes brings about a number of acute complications, such as cardiovascular diseases, cerebrovascular diseases, renal disorders, and obesity [5]. In addition, people with diabetes experience deterioration when it comes to the healing of serious wounds. Such individuals are susceptible to the development of acute, unhealable diabetic foot ulcers (DFUs). Wound healing is modulated by several mechanisms as well as physiological processes [6]. The process is complex, as it consists of a variety of different linked stages, which include hemostasis, inflammation, proliferation, and remodeling [7]. Typically, when an individual is wounded, the initial phase of hemostasis starts instantly through vascular constriction and fibrin clot formation. During the wound-healing process, pro-inflammatory cytokines and growth factors are produced by the clot and the surrounding wound tissue. After the control of bleeding, inflammatory cells migrate into the wound (chemotaxis), which facilitate the inflammatory phase, involving the sequential entry of neutrophils, macrophages, and lymphocytes. This is usually followed by the proliferative phase, which overlaps with the inflammatory phase and involves epithelial proliferation and movement above the provisional matrix of the wound; this is known as re-epithelialization. Then, the final remodeling sets in, and it is possible for this stage to happen over years. It is characterized by the regression of numerous newly produced capillaries, in order to return the wound’s vascular density to normal [8]. On the other hand, when bacteria colonize and replicate within the wound, the process of wound healing is delayed.. The bacterial species that are often isolated from such wounds include *Staphylococcus aureus, Enterococcus faecalis, Pseudomonas aeruginosa*, and others. When topical antimicrobial agents such as silver sulfadiazine are used, they can reduce the risk of a wound becoming infected during the process of healing; yet, microbial resistance against such antibiotics brings immense public health challenges [7,9]. In this regard, ceftriaxone was developed as a third-generation cephalosporin with a high efficacy and low toxicity. However, progressive resistance has emerged due to its indiscriminate use; thus, nano-delivery strategies have been introduced to overwhelm such resistance [10].

Anti-microbial peptides (AMPs) are host defense peptides with a positive charge and are found in a diversity of life systems ranging from microorganisms to humans. Most AMPs are capable of killing microbial pathogens directly, although others can indirectly modulate the host’s defense mechanisms. Indeed, great effort is being made to convey AMPs into clinical practice due to the accelerated resistance development to conventional antibiotics worldwide [11]. Essentially, AMPs have a high affinity for bacterial membranes. The negative anionic phospholipids that make up bacterial membranes and the positively charged AMPs render AMPs highly selective. Thus, bacteria-specific AMPs are driven by negatively charged membranes, which further bind AMPs [12]. Among AMPs, apamin is a bioactive peptide that makes up 2–3% of bee venom and plays a vital role as an antimicrobial through various mechanisms against bacteria, viruses, and fungi [13,14].

Hydrogels are materials of wide usage in the soft tissue engineering of blood vessels, muscles, skin, and fats. Due to their insoluble hydrophilic structures, they can absorb exudates from wounds and enable oxygen diffusion to quicken healing. They are characterized by a well-hydrated 3D polymeric network and are capable of binding an amount of water exceeding their dry weight; hence, they maintain a high moisture level in the wound bed. It is possible to cast hydrogel networks into a variety of sizes and shapes because of their special physical features. Hence, hydrogel-based materials are the most appropriate material for covering skin wounds [15]. It is possible to incorporate apamin into the hydrogels by mixing in polymer [16], using charged interactions [17], conjugating with the polymers utilized to form the hydrogel [18], or using the apamin as a self-assembling peptide for the formation of the hydrogels [19]. Due to the high water content, hydrogels aid the retention of the peptide’s activity and disallow denaturation. They minimize peptide’s enzymatic degradation through protection within the polymeric networks of the hydrogel. Additionally, the loading of stimuli-responsive hydrogels with apamin offers microenvironment-sensitive peptide release to achieve controlled, targeted, and effectual therapy [20]. Interestingly, many nanoparticles possess their own antimicrobial activities, probably due to their small size, which allows them to diffuse into the bacterial cells, besides not being prone to bacterial resistance [21]. Hence, nanoparticles have been incorporated into wound dressings due to their antibacterial and wound-healing actions [22,23]. Therefore, the aim of current work was to enhance wound healing in diabetic rats by conjugating the anti-microbial peptide “apamin” (APA) with the broad-spectrum antibiotic “ceftriaxone” (CTX) into a nanocomplex.

## 2. Materials and Methods

### 2.1. Materials

Apamin (APA) was purchased from Jinlan Pharm-Drugs Technology Co., Ltd. (Hangzhou, China); ceftriaxone (CTX) was kindly gifted from Tabuk Pharmaceutical Mgf. Co. (Tabuk, Saudi Arabia); hydroxypropyl methylcellulose (HPMC) (2% solution, viscosity: 4000 cp, 86,000 g/mol), was purchased from Acros Organics (Morris Plains, NJ, USA); streptozotocin (STZ) was purchased from Sigma-Aldrich Corporate (St. Louis, MO, USA); and CENEMEB^®^ ointment was kindly gifted from Unicare for Cosmetics and Antiseptics (Riyadh, Saudi Arabia).

### 2.2. Methods

#### 2.2.1. CTX–APA Nanoconjugate Preparation and Optimization

CTX–APA nanoconjugates were prepared by utilizing a three-factor Box–Behnken design that included CTX concentration (mM, X_1_), incubation time (min, X_2_), and sonication time (min, X_3_) as independent variables, while the following responses were the dependent variables: particle size (PS, nm, Y_1_) and zeta potential (Table 1). As per the design, the software developed 17 formulations, including three replicates of center points (Table 2). The main effects of the variables on the studied responses were assessed via ANOVA at a 95% level of significance by means of the Design-Expert^®^ software, version 12 (Stat-Ease Inc., Minneapolis, MN, USA). The equations of the chosen sequential model for each response were created in terms of coded factors. Moreover, the desirability function was calculated to select the optimal formulation. The anticipated targets were set to minimize the particle size and maximize the extent of the zeta potential (Table 1).

#### 2.2.2. Characterization of the Prepared CTX–APA Nanoconjugation Formulations

##### Particle Size and Zeta Potential Measurements

A Malvern zetasizer, Nano ZSP, (Malvern Instruments Ltd., Malvern, UK), was used to estimate the average particle size and the zeta potential values for the seventeen formulations of the CTX–APA nanoconjugations. The particle size measurement was achieved using a dynamic light-scattering technique with noninvasive backscatter optics, while the zeta potential was measured using laser doppler micro-electrophoresis. The repetition of all sample measurements was in triplicate. 

##### Stability of the Prepared Optimized CTX–APA Nanoconjugate

The particle size stability of the optimized CTX–APA nanoconjugate was assessed by subjecting the dispersion to three freeze (−20 °C) and thaw (20 °C) cycles for 12 h each [24]. The dispersion was investigated for a change in the particle size using the same Nano ZSP as before, with the same conditions.

##### Transmission Electron Microscope Imaging of Optimized CTX–APA Nanoformulation

The prepared CTX–APA was morphologically characterized using transmission electron microscopy (TEM) (Model: JEOL-JEM-1011, JEOL-Tokyo, Japan). On a carbon-coated grid, a few drops of the prepared formulation were placed and left for about 3 min to improve the vesicle adsorption on the carbon film; then, the excess liquid was removed by a filter paper. One drop from a 1% aqueous solution of phosphotungstic acid was added, followed by examining the preparation under TEM with an acceleration voltage of 80 kV at the Regional Center for Mycology and Biotechnology (Al-Azhar University, Cairo, Egypt).

#### 2.2.3. Preparation of CTX–APA Nanoconjugate-Loaded Hydrogel Formulations

APA is slightly soluble in water (1 mg/mL), but it is soluble in 0.05 M acetic acid (5 mg/mL) [25]. An amount of 750 mg of APA was placed in a beaker with 200 mL of 0.05 M acetic acid over a magnetic stirrer for complete dissolution to prepare the optimized CTX–APA nanoconjugate formulation. Similarly, the complete dissolution of 10 mL of 0.05 M acetic acid and 206.25 mg of CTX in a beaker was achieved after stirring at room temperature. This was followed by adding the CTX solution to the APA solution, stirring for few seconds, then incubating for 82 min at room temperature. After that, the mixture was homogenized by a CF3 2EY water bath sonicator (Ultra-wave Ltd., Cardiff, UK) for 10 min. Then, the excess solvent was gradually removed via evaporation at 36 °C using a rotavapor (BUCHI labortechnik AG, Flawil, Switzerland). Finally, acetic acid (0.05 M) was added to adjust to the final volume (50 mL). 

The topical gel formulation was prepared by simple dispersion of HPMC (2% *w*/*v*) to the adjusted volume of the optimized CTX–APA nanoconjugate formulation while stirring until the formation of a homogenous mixture was obtained, indicating complete dispersion of the polymer. Three hydrogel formulations (APA-loaded, CTX-loaded, and plain hydrogels) were prepared in order to compare with the optimized CTX–APA nanoconjugate-loaded hydrogel formulation. The APA gel formulation was prepared by placing 750 mg of APA in a beaker with 200 mL of 0.05 M acetic acid over a magnetic stirrer until it was completely dissolved. The excess solvent was gradually removed by evaporation at 36 °C using a rotavapor (BUCHI labortechnik AG, Flawil, Switzerland). Then, the final volume was adjusted to 50 mL using 0.05 M acetic acid. HPMC (2% *w*/*v*) was added by stirring until there was an absence of any lumps or precipitate. To prepare the CTX gel formulation, 206.25 mg of CTX was placed in a beaker with 50 mL of 0.05 M acetic acid and stirred at room temperature until it was completely dissolved. Again, HPMC (2% *w*/*v*) was added while stirring until the formation of a homogenous mixture. To prepare the plain hydrogel, a volume of 50 mL of 0.05 M acetic acid was placed alone in a beaker. HPMC (2% *w*/*v*) was added by stirring until complete dispersion of the polymer was achieved. All prepared formulations were kept at 4 °C until further characterization. 

#### 2.2.4. In Vivo Animal Handling

Male Wistar rats weighing between 190 and 230 g were attained from the vivarium of the Faculty of Pharmacy, King Abdulaziz University (KAU). Animals were kept at a relative humidity range of 40–70% and a temperature of 22 ± 2 °C, under a 12/12 h light–dark cycle. The animal handling procedures were approved in advance by the Committee of Research Ethics, Faculty of Pharmacy, KAU (Reference # PH-126-41).

#### 2.2.5. Skin Irritation Test

OECD guideline 404 was used to carry out a skin irritation test on five rats for the safety test. This involved careful shaving of a 2 × 2 cm area of hair on the back of each rat 24 h prior to treating them. An amount of 0.5 g of each gel was applied to the 2 × 2 cm area, then covered with gauze and tape. Four hours later, water was used to wash off the gels, followed by the observation of edema and erythema daily for 14 days after the removal of the gels. A scoring method ranking severity (0 to 4 scale) was used to assess edema and erythema. Rats with a 0 score were subjected to the same previous procedures [26].

#### 2.2.6. Induction of Diabetes

Sixty rats were deprived of food and had free access to water one night prior to the DM induction. A citrate buffer was prepared by combining 2 parts 0.1 M sodium citrate with 3 parts 0.1 M citric acid to produce the 0.1 M citrate buffer (pH adjusted to 4.0 using 1 N NaOH). The citrate buffer was added to streptozotocin (STZ), a toxin with a specificity for insulin-producing cells, in a sterile tube in a biosafety hood right before use to a final concentration of 5 mg/mL on ice. A single 60 mg/kg IP injection of STZ was given to each rat and food was placed in the cages thereafter. A glucometer was used to measure blood glucose levels. The rats were assessed for two weeks after injecting them, and those with blood glucose levels higher than 250 mg/dL were considered diabetic and included in the study.

#### 2.2.7. Wound Excision

A mixture of ketamine/xylazine (100/10 mg/kg) was used to anesthetize the diabetic rats. Ethanol (70%) was used to disinfect their shaved skin at the dorsal surface. This was followed by the excision of a 2 × 2 cm area of skin from the epidermal to subcutaneous layers as well as the underlying connective tissue on the dorsal surface of the rat. After disinfecting the wounds, pain reduction was achieved by an injection of lidocaine hydrochloride (2%) with 1:80,000 epinephrine subcutaneously, at a dose of 4.4 mg/kg near the excision area. 

Each diabetic rat was placed in a separate cage, and was given standard feed pellets and water ad libitum. The cages were randomly divided into 6 groups and labeled accordingly (*n* = 10 per group). The groups were considered as Group 1: untreated control; Group 2: hydrogel-treated rats (vehicle); Group 3: CTX loaded in hydrogel; Group 4: APA loaded in hydrogel; Group 5: CTX–APA loaded in hydrogel; and Group 6: positive control rats treated with CENEMEB^®^ ointment, Tabuk Pharmaceutical Manufacturing Company (with *β*-Sitosterol as the active ingredient, in a base of beeswax and sesame oil). 

All treatments were sustained for 3 weeks. Wound diameters (WD) were measured and wounds were photographed on days 0, 3, 7, 10, 14 and 21. On day 10, four rats per group were sacrificed by decapitation, and the wounded skin was dissected out and kept in 10% neutral formalin. On day 21, all remaining animals were sacrificed by an IP injection of pentobarbital (800 mg/kg) [27], followed by dissecting the skin in the wound area. A part of each skin was kept in 10% neutral formalin and the other part was instantly frozen in liquid nitrogen and stored at −80 °C for further analyses. 

#### 2.2.8. Wound Measurement

The percentage of wound contraction was determined through the following formula:

Wound contraction % = (WD on day 0 − WD on day 21)/(WD on day 0) × 100

#### 2.2.9. Preparation of Tissue Homogenate

The first step was the careful rinsing of the obtained skin specimens using ice-cooled saline, followed by gently blotting the skin between filter papers and weighing. The next step was the preparation of 10% (*w*/*v*) of homogenates in phosphate-buffered saline (PBS, 50 mM potassium phosphate, pH 7.4, ice-cooled) before centrifugation at 3000 rpm for 20 min at 4 °C. An estimation of malonaldialdehyde (MDA) was performed in the supernatant, in addition to reduced glutathione (GSH) content, superoxide dismutase (SOD) activity, and catalase (CAT) activity as antioxidant biomarkers. This allowed for the further assessment of the hydroxyproline content.

#### 2.2.10. Histological Examination

Tissues were fixed in a 10% neutral formalin solution for 24 h, then dehydrated and embedded in paraffin, followed by the sectioning of tissues in a paraffin block (5 µm thickness). The rehydration of tissues took place after dewaxing. Hematoxylin and eosin (H&E) were used to stain certain sections and Masson’s trichrome (MT) was used to stain the remaining sections. Histological examinations were performed by a pathologist without prior knowledge of the treatment groups. Based on the degree of granulation in the tissue, re-epithelization, collagen deposition, inflammatory cell infiltration, and the wound-healing phase (I, II, or III) on day 10, sections were given scores ranging from − to +++. 

#### 2.2.11. Biochemical Analyses

Assessments of the total protein, CAT, SOD, GSH, and MDA were performed using biochemical kits (catalog # 10009055, 703002, 706002, 707002, and 701780, respectively; Cayman^®^ Chemical, Ann Arbor, MI, USA). The hydroxyproline content in the skin was determined using an ELISA kit (catalog # ab222941, Abcam^®^, Cambridge, UK), based on the instructions provided in the product pamphlet. 

#### 2.2.12. RT-qPCR of Excised Tissue

An ultrasonic probe was used to homogenize the tissues from each rat on day 21. The extraction of RNA was performed using the nucleic acid extraction kit obtained from NucleoSpin^®^ (Macherey-Nagel GmbH & Co. KG, Duerin, Germany). A dual-wavelength spectrophotometer (Shimadzu, Spectrophotometer, Japan) was used to obtain the RNA concentration and the estimated purity of the A260/A280 ratio. Reverse transcription was performed using a High-Capacity cDNA Reverse Transcription Kit from Applied Biosystems (Foster City, CA, USA) for the construction of a cDNA library. This was followed by the completion of PCR amplification with a Taq PCR Master Mix Kit (Qiagen, Valencia, CA, USA), the Col1A1 (NM_053304.1) primer, and GPADH (NM_017008.4) as a housekeeping gene. The forward/reverse nucleotide sequences of GAPDH, Col1A4, and Col1A1 were CCATTCTTCCACCTTTGATGCT/TGTTGCTGTAGCCATATTCATTGT, TGGTCTGCAAGGAATGCCTGGA/TCTTTCCCTGGGACACCATCAG, and ATCAGCCCAAACCCCAAGGAGA/CGCAGGAAGGTCAGCTGGATAG, respectively. The expression of the data in the cycle threshold (Ct) took place following the RT-PCR run. A delta-delta Ct (ΔΔCt) calculation formed the basis for determining the relative quantitation (RQ) of Col1A1 with respect to GAPDH.

#### 2.2.13. Immunohistochemical Assessments

The detection of inflammatory cytokines (IL-6 and TNF-α) in tissue slices was performed using the immunohistochemical (IHC) technique by labeling with horseradish peroxidase (HRP), which is capable of reacting with certain substrates to produce a brown coloration in tissue. The tissue slices (4 µm thickness) underwent a brief immunohistochemical staining with the primary rabbit antibodies to IL-6, TNF-α, TGF-β1, HIF-1α, and VEGFA (catalog # ab9324, ab220210, ab215715, ab228649, and ab231260, respectively; Abcam^®^, Cambridge, UK). Image analysis software (ImageJ, 1.48a, NIH, Bethesda, MD, USA) was used to carry out image quantitation by using the optical density (OD) of brown-stained cells across 6 different fields for every rat section.

#### 2.2.14. Statistical Analysis

The data are displayed as means ± SD. The statistical analyses were performed using one-way analysis of variance (ANOVA) followed by Tukey’s post hoc test. The level of significance was accepted as *p* < 0.05. After carrying out the analyses, the figures were sketched using GraphPad Prism software, version 8.00 (GraphPad Software, La Jolla, CA, USA).

## 3. Results

### 3.1. Box–Behnken Design Statistical Analysis

#### 3.1.1. Model Fit Statistics

Table 3 compiles the findings of the model fit statistics for the measured PS and ZP. According to the highest computed R2 and lowest PRESS among the investigated sequential models, PS fitted the quadratic model, while ZP fitted the linear model. For both responses, the adjusted and predicted R2 coincided reasonably, where the difference between them was less than 0.2. In addition, the adequate precision values were markedly higher than the desirable value of 4 (16.36 and 20.65 for PS and ZP, respectively), indicating a good signal-to-noise ratio. Thus, the selected models were considered adequate for the investigation of the design space.

**Figure 1:**
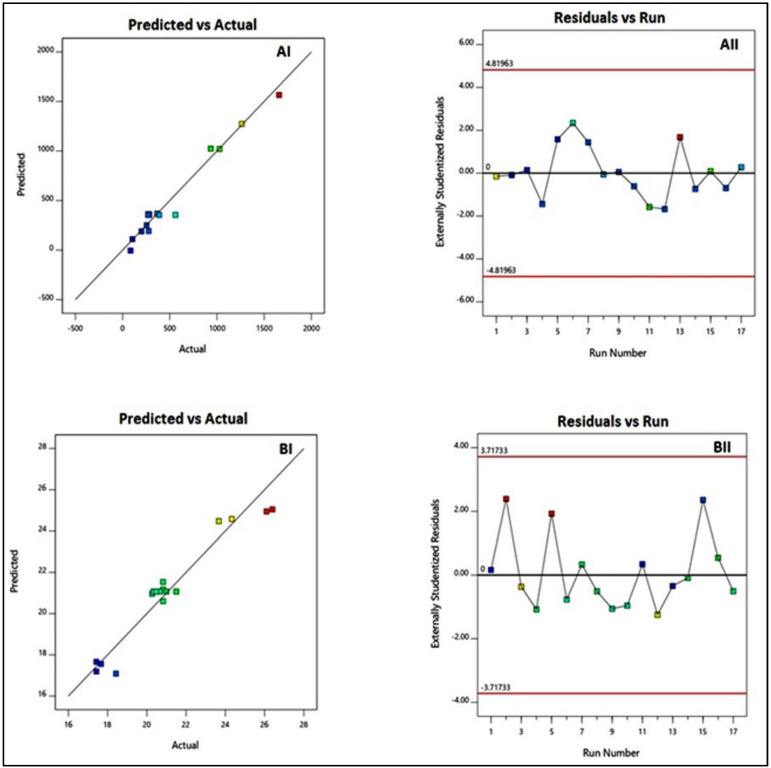
Diagnostic plots for particle size (**A**) and zeta potential (**B**) of CTX–APA nanoconjugates. (**I**) indicates the plots of predicted vs. actual values; (**II**) indicates the plots of externally studentized residuals vs. run number.

The diagnostic plots, as shown in Figure 1, were created to determine the goodness of fit for the best-fitting sequential models. The predicted vs. actual particle size plots (Figure 1AI,BI) show a good linear pattern that indicates a good correlation between the expected and actual values for all responses. Furthermore, the residual vs. run plots (Figure 1AII,BII) display randomly distributed points in-between the limits, which advocates the absence of any lurking variable that could affect the measured responses [24,28].

#### 3.1.2. Variable Influence on Particle Size (Y_1_)

The prepared CTX–APA nanoconjugates showed wide variation in the particle size, ranging from 103.9 ± 2.3 to 1658.7 ± 57.8 nm (Table 2). The ANOVA (Table 4) for the particle size revealed the relevance of the quadratic model, with an F-value of 21.99 (*p* = 0.0030). The equation for the best-fitting quadratic model was produced in terms of the coded factor (Equation (1)).

Y_1_ = 355.30 + 528.11 X_1_ − 13.59 X_2_ + 72.52 X_3_ − 111.11 X_1_X_2_ + 199.57 X_1_X_3_ + 15.69 X_2_X_3_ + 368.10 X_1_^2^ − 102.89 X_2_^2^ + 41.63 X_3_^2^ (1)

**Figure 2:**
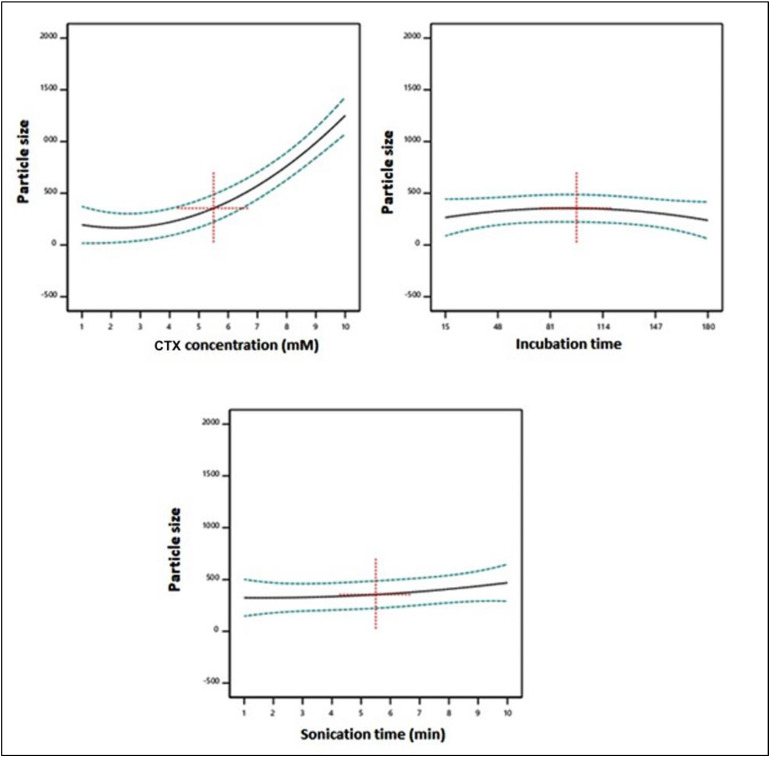
Main effects of the investigated independent variables on the particle size of CTX–APA conjugates.

**Figure 3:**
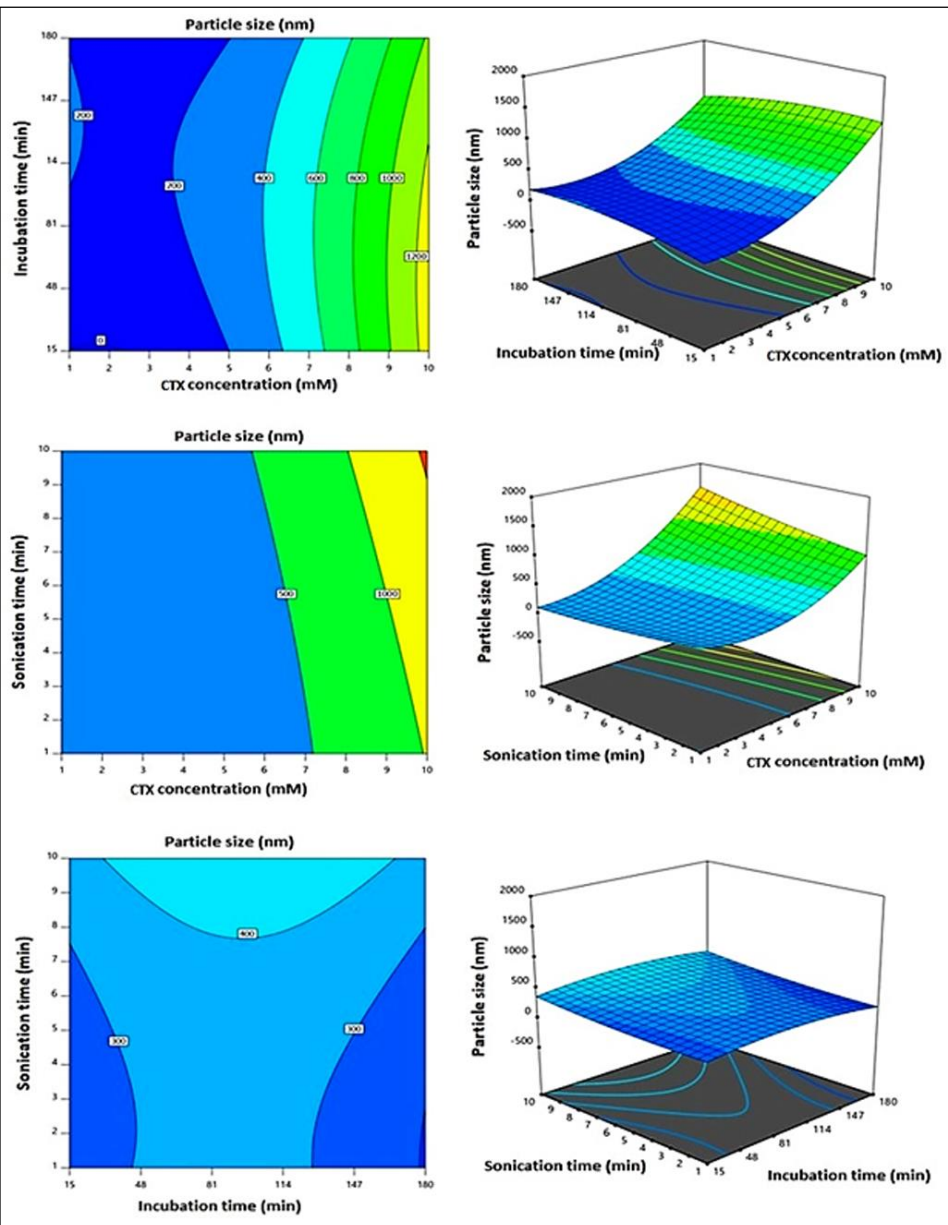
The 2D contour and 3D surface plots for the effect of the interaction between independent variables on the particle size of CTX–APA conjugates.

The linear term X_1_, corresponding to the CTX concentration, and its corresponding quadratic term X_1_^2^ had a major influence on particle size (*p* < 0.0001 and *p* = 0.0005, respectively). Furthermore, the interaction of X_1_X_3_, conforming to the interaction between the CTX concentration and the sonication time, was also significant (*p* = 0.0153). Figure 2 demonstrates the main effects of the investigated variables on the particle size, while Figure 3 displays the two-dimensional contour and the three-dimensional surface plots for the interactions between them. It was evident that the size was directly proportional to the CTX concentration, i.e., the size increased at higher CTX concentrations. The positive sign and the highest magnitude of coefficient of X_1_ among the linear terms in the equation support this observation.

#### 3.1.3. Variable Influence on Zeta Potential (Y_2_)

The prepared CTX–APA conjugates exhibited zeta potentials ranging from 18.4 ± 0.7 to 26.1 ± 1.1 mV (Table 2). The ANOVA for the zeta potential (Table 5) revealed the relevance of the linear model, with an F-value of 58.12 (*p* < 0.0001). The equation for the best-fitting linear model was produced in terms of the coded factor (Equation (2)).

Y_1_ = 21.07 − 3.69 X_1_ − 0.1813 X_2_ + 0.2862 X_3_ (2)

**Figure 4:**
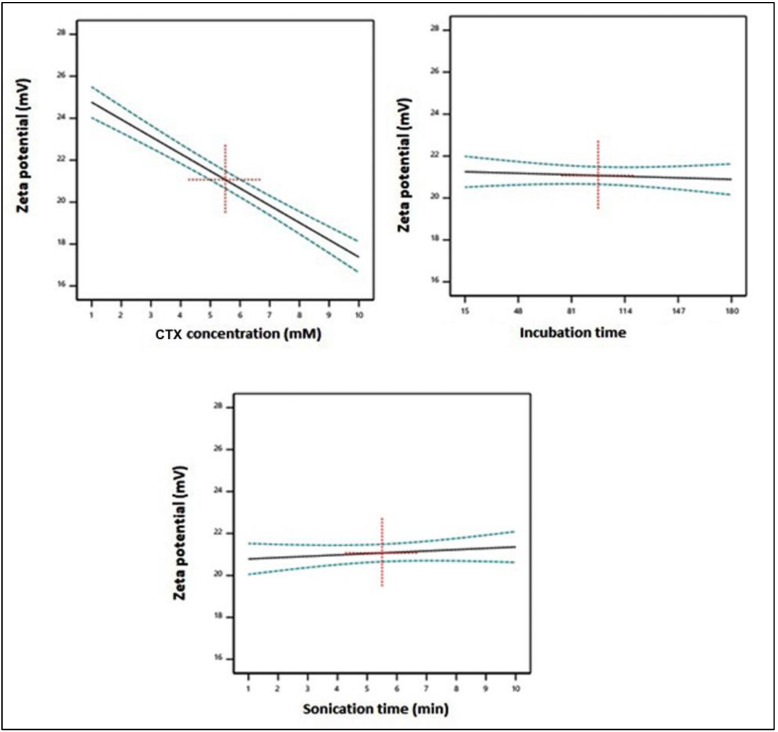
Main effects of the investigated independent variables on the zeta potential of CTX–APA conjugates.

**Figure 5:**
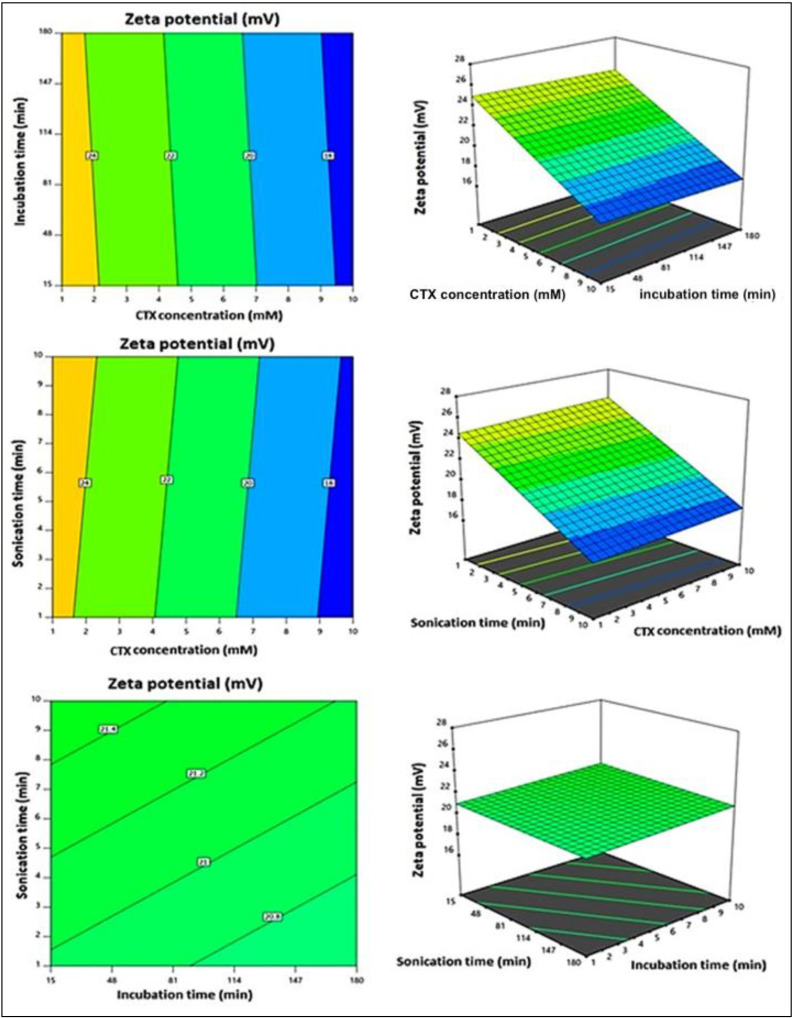
The 2D contour and 3D surface plots for the effect of the interaction between independent variables on the zeta potential of CTX–APA conjugates.

The linear term X_1_, corresponding to the CTX concentration, exhibited a significant impact on particle size (*p* < 0.0001). Figure 4 illustrates the main effects of the investigated variables on the zeta potential, while Figure 5 displays the two-dimensional contour and the three-dimensional surface plots for the interactions between them. According to the illustrations, the zeta potential decreased significantly with increasing CTX concentration. The negative sign of the X_1_ coefficient supports such observation. This could be credited to the negative charge of the drug.

### 3.2. Optimization

Following the limitations formerly set for the particle size and zeta potential, the optimized levels of the formulation variables were expected with an overall desirability of 0.857. The expected levels were 1.00 mM for the CTX concentration, 66.40 min for the incubation time, and 10.0 min for the sonication time. The optimized formulation was prepared and assessed as shown in Table 6. The relatively low percentage of error between the anticipated and measured responses proved the effectiveness and validity of the optimization process. It is worthy to note that the amount of error is usually related to the experiment, and generally a margin of 10% is acceptable; however, in some cases a higher percentage can be accepted. In our case concerning the nanoconjugate size, the observed size could be slightly higher than the predicted one, owing to possible aggregation.

Regarding stability, the optimized formula showed no significant changes (*p* < 0.05) to particle size after repeated freeze-thaw cycles, which indicated the stability of the prepared optimized nanoconjugate.

### 3.3. TEM Investigation of Optimized CTX–APA

**Figure 6:**
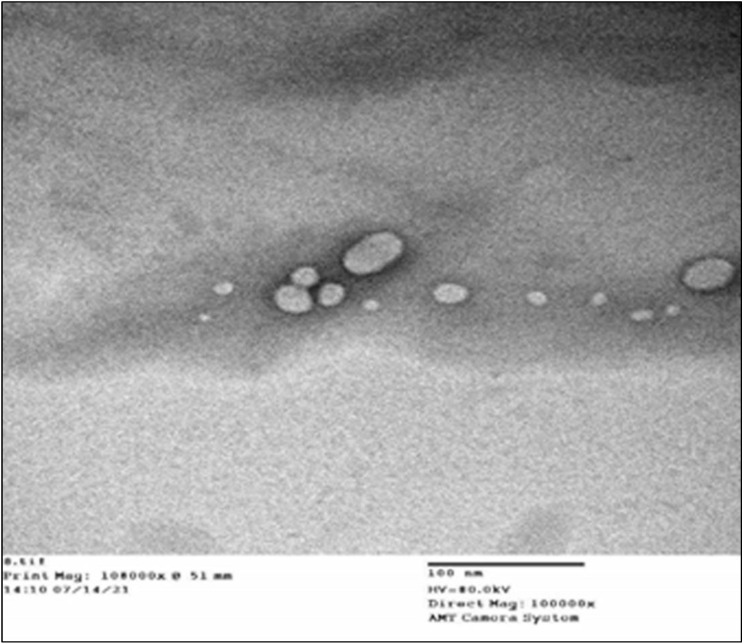
TEM image of optimized CTX–APA nanoconjugates.

The TEM results (Figure 6) displayed spherical bodies with a size range analogous to the size obtained by using the nanosizer. Earlier studies have indicated that particle sizes of about 100 nm attain maximum cellular uptake [23,24]. Hence, the CTX–APA characterization showed a promising formula with respect to the particle size and zeta potential.

### 3.4. Assessment of Wound Healing

**Figure 7:**
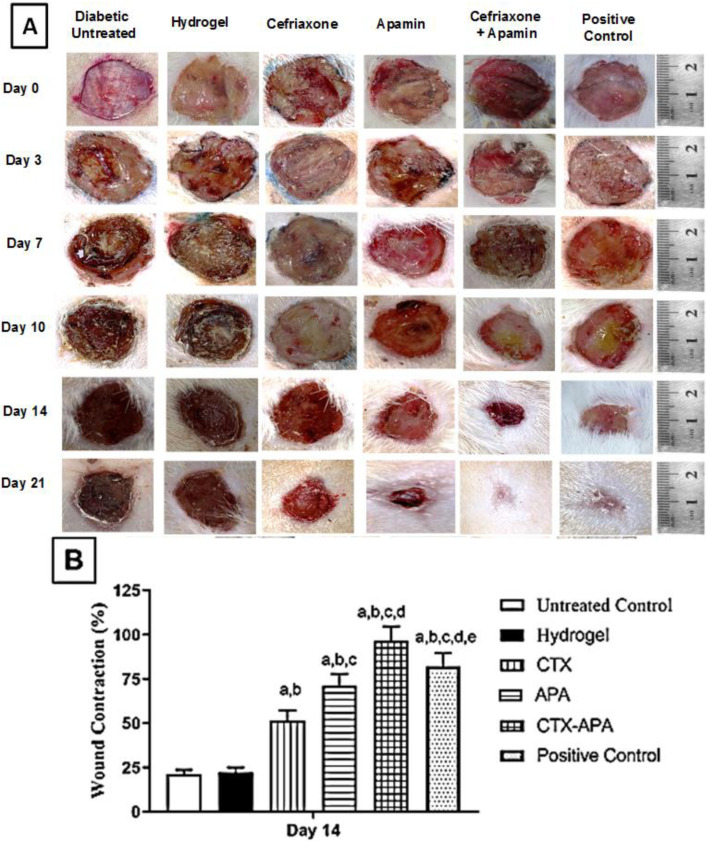
Panel (**A**) shows photographs of wound closure in diabetic rats treated with topical applications of the optimized CTX–APA-loaded hydrogel. Panel (**B**) shows the effects of different preparations on wound closure. Data are displayed as means ± SD (*n* = 6). Statistical analyses were performed using one-way ANOVA followed by Tukey’s test. ^a^ Significantly different from untreated control group. ^b^ Significantly different from hydrogel. ^c^ Significantly different from CTX group. ^d^ Significantly different from APA group. ^e^ Significantly different from CTX–APA. For all analyses, the significance level was set to *p* < 0.05.

The wounds reduced slightly until day 3, as shown by the topical applications of the CTX- and APA-loaded hydrogels. The application of the optimized CTX–APA-loaded hydrogel enabled faster wound healing on day 7 relative to the hydrogel control group and the untreated group. The wounds exposed to the optimized CTX–APA exhibited almost complete healing by day 21. The prepared combined formula exhibited greater healing activities relative to the positive control group (Figure 7A). When the wound contraction percentage was assessed, the animals treated with the optimized CTX–APA exhibited remarkably higher wound contractions (Figure 7B).

### 3.5. Histopathological Assessment of Wound Tissues Stained with H&E and MT

**Figure 8:**
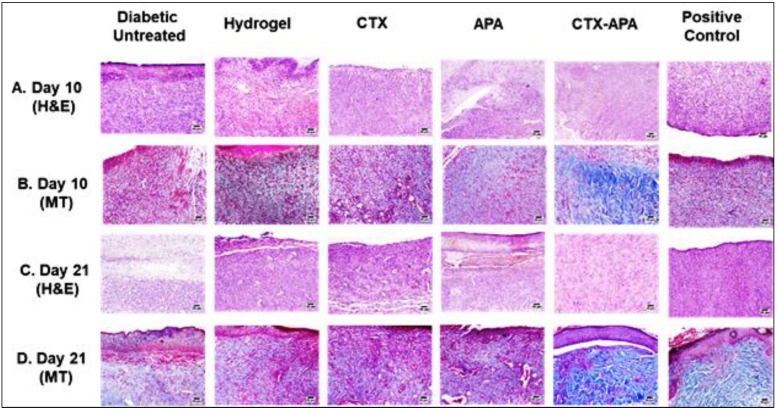
Histopathological effect of CTX–APA on wound healing of diabetic rats. Panels (**A**,**B**) denote skin sections from wounded areas collected on day 10 and stained with H&E and MT, respectively. Panels (**C**,**D**) denote skin sections from wounded areas collected on day 21 and stained with H&E and MT, respectively.

Days 10 and 21 featured the detection of poor wound healing in the vehicle-treated control (hydrogel) and diabetic untreated control animals. There was evidence of extremely inflamed, less organized granulation without a complete new epidermal covering, which seemed restricted to the wound edges only—related to the infiltration of heavy neutrophils. The CTX- and APA-loaded hydrogel groups showed signs of improved healing, revealing moderately inflamed granulation tissue with many newly produced blood vessels. Additionally, epidermal remodeling was detected in various sections examined on day 21. Some fields exhibited organized tissue. The optimized CTX–APA-loaded hydrogel group exhibited the greatest degree of wound healing on day 10 after the wounding. The examined sections revealed an almost total epidermal remodeling, together with evidence of keratinization on day 21. There was healthy organized tissue in the wound gap, with a slight inflammatory reaction and numerous collagenous matrixes. Improved wound healing was observed in the examined wounds of different animals for the positive control group. Mildly to moderately inflamed granulation tissues were observed in a number of examined sections in the wound gap. There was no epidermal covering in a number of sections, which was related to the persistence of neutrophilic infiltration and the necrotic crust (Figure 8A,C). The staining of skin sections using Masson’s trichrome stain (MT) helped with assessing collagen deposition as a marker of wound healing. A high degree of collagen deposition and numerous collagen fibers in the wound gap indicated a speedy rate of wound healing for the CTX–APA group (Figure 8B,D).

Table 7 shows the results of an additional semi-quantitative assessment of the histological findings. As shown in the table, diabetic rats treated with the combined formula displayed the highest rate of healing in terms of the highest collagen deposition, with most of the skin tissues being in the proliferation phase (Phase II) or re-modeling phase (Phase III) of healing.

### 3.6. Effect of Optimized CTX–APA on Oxidative Status on Day 21

The data in Table 8 indicate that the tissues treated with CTX–APA or the positive control experienced a significant lowering of the MDA content by 47.4 and 33.6%, respectively, as compared to the hydrogel-treated controls. In addition, the CTX–APA group exhibited the uppermost antioxidant activity as compared to the hydrogel and positive control groups. The CTX–APA treatment significantly boosted the SOD, CAT, and GSH in the wound tissue homogenates by about 70, 350, and 82%, respectively, as compared to the hydrogel-treated control.

### 3.7. Effect of CTX–APA on Inflammation on Day 21

**Figure 9:**
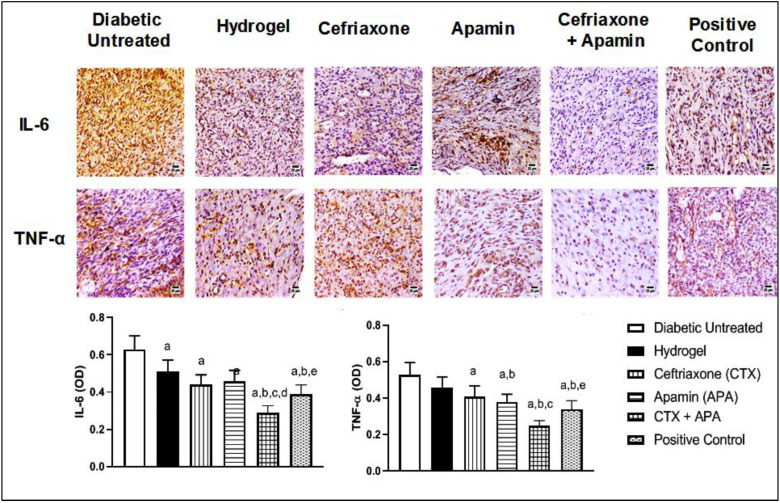
Effect of CTX–APA on inflammatory cytokine IL-6 and TNF-α content in wounded skin of diabetic rats. Data are shown as means ± SD (*n* = 6). One-way ANOVA followed by Tukey’s test was performed and the significance level was set to *p* < 0.05 for all analyses. ^a^ Significant difference from diabetic untreated group. ^b^ Significant difference from hydrogel. ^c^ Significant difference from CTX. ^d^ Significant difference from APA. ^e^ Significant difference from CTX–APA.

The treatment of diabetic rats with CTX–APA or the positive control preparation significantly reduced IL-6 in wound tissues by 54 and 38% compared to the untreated control, respectively. Likewise, the TNF-α content in wound tissue was significantly lowered by 52 and 35% compared to the untreated control group, respectively, as shown in Figure 9.

### 3.8. Effect of CTX–APA on Collagen Formation on Day 21

**Figure 10:**
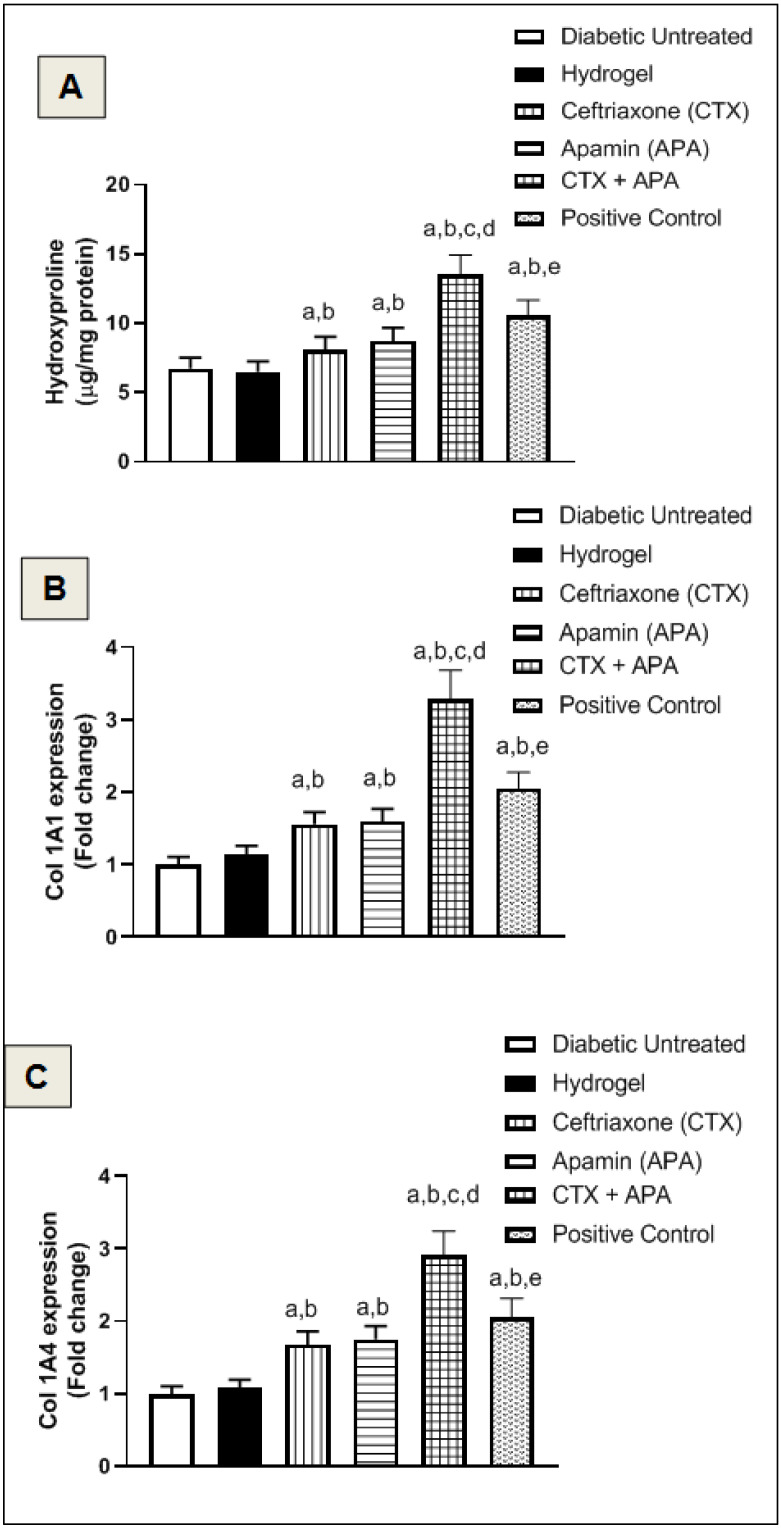
Effect of CTX–APA on collagen formation in wounded skin of diabetic animals. Panel (**A**): hydroxyproline content, Panel (**B**): relative Col 1 mRNA expression, and Panel (**C**): relative Col 1A4 mRNA expression. Data are demonstrated as means ± SD (*n* = 6). One-way ANOVA followed by Tukey’s test was performed and the significance level was set to *p* < 0.05 for all analyses. ^a^ Significant difference from diabetic untreated group. ^b^ Significant difference from hydrogel. ^c^ Significant difference from CTX. ^d^ Significant difference from APA. ^e^ Significant difference from CTX–APA.

Both APA and CTX–APA significantly boosted the hydroxyproline skin content by 25 and 100% compared to the hydrogel-treated control, respectively, as indicated in Figure 10A. As well, both preparations upregulated the relative mRNA expression of Col1A1 by approximately 180 and 80% compared to the vehicle-treated control, respectively (Figure 10B,C).

### 3.9. Effect of CTX–APA on Immunohistochemical Expression of TGF-β1, HIF-1α, and VEGF on Day 21

**Figure 11:**
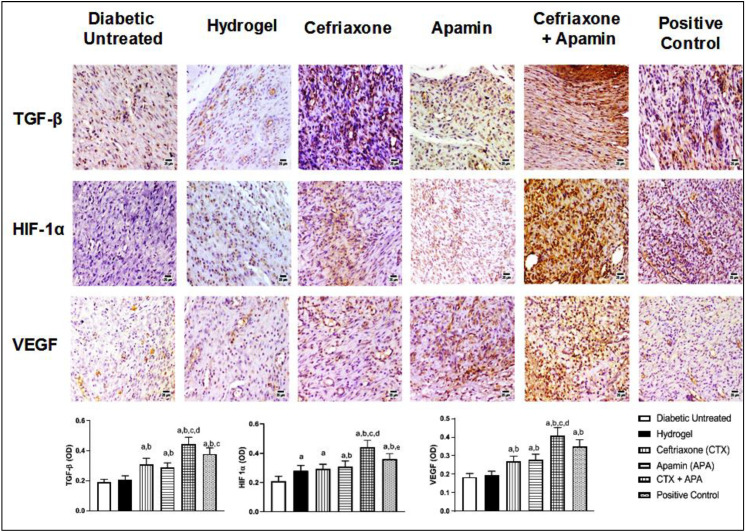
Effect of CTX–APA on expression of tumor growth factor-beta-1 (TGF-β1) (upper panel), hypoxia-inducible factor 1-alpha (HIF-1α) (middle panel), and vascular growth factor (VEGF) (lower panel) as shown by immunohistochemical staining. Data in the rightmost column are demonstrated as means ± SD (*n* = 6). One-way ANOVA followed by Tukey’s test was performed and the significance level was set to *p* < 0.05 for all analyses. ^a^ Significant difference from diabetic untreated group. ^b^ Significant difference from hydrogel. ^c^ Significant difference from CTX. ^d^ Significant difference from APA. ^e^ Significant difference from CTX–APA.

The immunohistochemical expression of the fibrotic marker “TGF-β1” as well the angiogenic markers “HIF-1α” and “VEGF” was utilized to determine the molecular processes underlying wound healing. Figure 11 illustrates the TGF-β1 expression in the wounds of untreated and hydrogel-treated controls. The treatment with CTX–APA triggered a significant rise in the TGF-β1 expression (by about 130%) compared to the untreated controls. In addition, the treatment with the combined preparation and the positive control obviously enhanced the HIF-1α expression by 57 and 29.5%, respectively, compared to the untreated and hydrogel-treated groups. Similarly, CTX–APA and the positive control preparation exhibited a noticeable increase in VEGF expression in comparison with the untreated and vehicle-treated controls, with the OD of the positively stained areas enhanced by about 121 and 89%, respectively, as compared to the untreated controls.

## 4. Discussion

The process of wound healing in uncontrolled diabetes is slow, and inefficient management can bring about complications [29]. The current study investigated the wound-healing features of a new CTX–APA nanocomplex formulation in streptozotocin-induced diabetic rats, and aimed to assess possible synergistic interactions between these two compounds when the wound-healing process was enhanced [30,31]. The particle size of the CTX–APA nanocomplex was ~100 nm (60.27 ± 2.8 nm), while its zeta potential of 23.67 ± 2.66 mV was considered an optimal value for particle stability (~30 mV). In line with past research reports, the achievement of maximum cellular uptake was possible with the particle size of the nanocomplex [31,32]. The enhancement of cellular translocation using nanobodies with a positive charge has been proven, and this likewise made the optimized formulation more stable relative to the drugs when individually considered [33]. However, a noteworthy point here is that one of the crucial measures for colloidal dispersion stability is the zeta potential, which impacts many features of the formulation such as performance and efficiency, thereby sustaining a more stable product. The CTX–APA nanocomplex formulation, which contained CTX and APA (1:1 ratio), was utilized in an in vivo model of an acute wound (diabetic rats) in order to test its preclinical effectiveness.

The CTX–APA nanocomplex formulation gains support from the previously reported ability of CTX to enhance wound healing in diabetic rats [34]. Further, CTX-entrapped liposomes significantly expedited the healing of *E. coli*-contaminated wounds in rats [35]. In addition, APA’s beneficial effects are consistent with the documented wound-healing activities of bee venom. It has been reported that APA brings about accelerated curative effects and could be applied as a new potential treatment for wound repair [36]. Further, it gains indirect support by the notion that mellitin, another related bee venom, enhances wound healing [34,37]. In addition, apamin has been shown to enhance the regeneration of injured cortical neurons in vitro [38].

The mechanisms of impaired healing responses are not fully characterized. However, accumulating evidence indicates a role of oxidative stress in the pathogenesis of the delayed healing of wounds [39]. This is of particular significance in diabetes [40]. Therefore, we have explored the potential of an optimized CTX–APA formula to combat the oxidative stress induced by wounding. Fortunately, the combined formula showed significant antioxidant properties. Our data are consistent with the reported antioxidant activities of APA. It has been reported to show potent activity against oxidative stress in a murine model of kidney injury, as it prevented the accumulation of 4-hydroxynone and MDA as well as glutathione depletion [41]. In addition, APA antioxidant activity was observed in cell-free systems, as it exhibited 2,2 diphenyl picrylhydrazyl (DPPH) scavenging activity, inhibition of thiobarbituric acid reactive species (TBARS), and β-carotene bleaching inhibition [42]. On the other side, CTX has also been shown to possess antioxidant activity, as it ameliorated all the markers of oxidative stress of deltamethrin-induced nephrotoxicity in rats [43]. Moreover, the antioxidant properties of CTX have been highlighted experimentally. It attenuated oxidative damage and restored reduced levels of endogenous antioxidant enzymes in animal models of neurodegenerative diseases [44,45]. In addition, CTX ameliorated oxidative stress in the hippocampus of pentylenetetrazole-kindled rats [46]. Thus, it can be suggested that the enhanced antioxidative properties of the optimized formula participated in the observed expedition of wound healing in diabetic rats.

Inflammation is considered a non-specific immune reaction involving the degeneration of tissue, which usually resolves after the infiltrated leukocytes revert to their pre-inflammatory conditions. A chemotactic response in leukocytes by inflammatory cytokines enhances the inflammation phase [47]. Therefore, inflammation represents the first phase of wound healing. Our data indicate that the CTX–APA treatment significantly expedited the inflammatory phase, as indicated by the reduced expression of IL-6 and TNF-α. This is in line with the reported anti-inflammatory activity of CTX. In a rat model of traumatic brain injury, CTX attenuated the consequences of the injury and exhibited anti-inflammatory activities [48]. In addition, it attenuated cerebrospinal fluid inflammation in experimental pneumococcal meningitis [49]. Further, CTX prevented glutamate-mediated neuro-inflammation in an experimental model of parkinsonism in rats [45]. This is in addition to the known anti-inflammatory properties of bee venom that were suggested to participate in the venom’s wound-healing activities [36]. APA is the second most important active peptide in bee venom and has been reported to show anti-inflammatory activity by antagonizing the activation of macrophages, and consequently, the inhibition of the PDGF-BB-induced phosphorylation of Akt and Erk1/2 and vascular smooth cell proliferation and migration [50]. In BV2 and rat primary microglial cells, APA inhibited LPS-induced inflammatory responses via regulating the SK channels and TLR4 signaling pathways [51]. Experimental animal studies have also highlighted potent anti-inflammatory actions of APA. In mice, it suppressed inflammation in a model of gouty arthritis [52]. In addition, it showed anti-inflammatory effects in lipopolysaccharide-induced acute kidney injury [41]. Collectively, the aforementioned findings tend to support the anti-inflammatory effects of both CTX and APA that contributed to the improved wound healing observed after CTX–APA application.

Remodeling is the last, albeit critical, stage of wound healing in which the tissue integrity is restored. It is characterized by the generation of new epithelial cells and scar formation [53]. In this regard, collagen is highly involved and has been reported to have advantageous effects in wound healing [54]. In the current study, hydroxyproline and the mRNA expression of Col 1A1 and Col 1A4 were enhanced by CTX and APA. This is consistent with the increased collagen deposition observed in Masson’s trichrome-stained skin sections. In addition, these findings fit very well with the expedited and complete wound healing observed in CTX–APA treated animals. This notion is supported by the reported collagen-enhancing properties of CTX in diabetic wounds [34]. In addition, indirect support of APA pro-collagen activities during wound healing is provided by the abilities of the related bee venom mellitin [37]. This is also consistent with the detected enhancement of TGF-β expression in this study. TGF-β 1, a multifunctional cytokine [55,56], plays an important role in all stages of wound healing through alleviating inflammation, stimulating granulation tissue formation, and attracting fibroblasts and myofibroblasts into the wounded area [57]. The formation of new blood vessels represents another critical event of wound healing. This angiogenesis occurs throughout all stages of wound repair [58]. HIF-1 has been reported to be involved in the process of angiogenesis through wound healing [59]. Further, VEGF is a strong stimulus for wound healing through angiogenesis, in addition to collagen deposition and epithelialization [60]. In general, bee venom has been reported to enhance angiogenesis via increasing the VEGF expression [36]. These data support our findings regarding the ability of both CTX and APA to boost VEGF expression. Collectively, the CTX–APA nanoformulation significantly expedited wound healing in diabetic rats.

## 5. Conclusions

The present study highlights the therapeutic potential of a novel CTX–APA nanoconjugate in hydrogels for the acceleration of wound healing in diabetes. The optimized nanocomplex, with respect to particle size and zeta potential, possessed greater wound-healing properties compared to all the other experimental conditions following 21 days of daily topical application. This effect could be attributed to its ability to counteract oxidative stress and inflammation. The CTX–APA nanocomplex was also found to enhance the process of angiogenesis, which has an important role in tissue recovery. Overall, these findings could represent a novel pharmacological tool to be used in vivo to accelerate delayed wound healing in diabetes.

## Figures and Tables

**Table 1:** Independent variables and responses of the experimental runs of CTX–APA conjugates according to Box–Behnken design.

Independent Variables	Levels		
	(−1)	(0)	(+1)
X_1_: CTX Concentration (mM)	1.0	5.5	10.0
X_2_: Incubation Time (min)	15.0	97.5	180.0
X_3_: Sonication Time (min)	1.0	5.5	10.0
**Responses**	**Desirability Constraints**		
Y_1_: Particle Size (nm)	Minimize		
Y_2_: Zeta Potential (mV)	Maximize		

Abbreviations: CTX, ceftriaxone; APA, apamin.

**Table 2:** The experimental runs and observed responses of CTX–APA conjugates according to Box–Behnken design.

Experimental Run Number	Independent Variables			PS ± SD (nm)	ZP ± SD (mV)
	CTX Concentration (mM)	Incubation Time (min)	Sonication Time (min)		
F-1	10.0	15.0	5.5	1263.6 ± 42.1	17.7 ± 0.9
F-2	1.0	97.5	10.0	103.9 ± 2.3	26.4 ± 1.2
F-3	1.0	180.0	5.5	199.6 ± 8.7	24.3 ± 0.9
F-4	5.5	15.0	10.0	280.7 ± 9.1	20.8 ± 0.8
F-5	1.0	15.0	5.5	84.6 ± 2.1	26.1 ± 1.1
F-6	5.5	97.5	5.5	559.8 ± 14.6	20.5 ± 0.7
F-7	5.5	180.0	1.0	276.0 ± 10.8	20.8 ± 0.6
F-8	5.5	180.0	10.0	364.9 ± 13.5	20.8 ± 0.8
F-9	5.5	15.0	1.0	254.6 ± 9.1	20.2 ± 0.7
F-10	5.5	97.5	5.5	283.5 ± 8.8	20.3 ± 0.8
F-11	10.0	180.0	5.5	934.2 ± 29.8	17.4 ± 0.5
F-12	1.0	97.5	1.0	270.5 ± 7.9	23.7 ± 1.1
F-13	10.0	97.5	10	1658.7 ± 57.8	17.4 ± 0.6
F-14	5.5	97.5	5.5	270.6 ± 11.6	21.0 ± 0.5
F-15	10.0	97.5	1.0	1026.9 ± 31.9	18.4 ± 0.7
F-16	5.5	97.5	5.5	274.6 ± 11.7	21.5 ± 0.7
F-17	5.5	97.5	5.5	387.9 ± 14.9	20.7 ± 0.6

Abbreviations: CTX, ceftriaxone; APA, apamin; PS, particle size; ZP, zeta potential.

**Table 3:** Fit summary statistics for the responses of CTX–APA conjugates according to the fitting model.

Response	Fitting Model	Sequential *p*-Value	Lack of Fit *p*-Value	R²	Adjusted R²	Predicted R²	PRESS
PS (nm)	Quadratic	0.0030	0.4674	0.9658	0.9219	0.7312	8.62 × 10^5^
ZP (mV)	Linear	<0.0001	0.1095	0.9306	0.9146	0.8650	15.96

Abbreviations: CTX, ceftriaxone; APA, apamin; PS, particle size; ZP, zeta potential; PRESS, predicted residual error sum of squares.

**Table 4:** ANOVA results for particle size of CTX–APA nanoconjugates.

Source	Sum of Squares	Degrees of Freedom	Mean Square	F-Value	*p*-Value
**Model**	3.098 × 10^6^	9	3.442 × 10^5^	21.99	0.0002
X_1_: CTX Concentration	2.231 × 10^6^	1	2.231 × 10^6^	142.54	<0.0001
X_2_: Incubation Time	1478.05	1	1478.05	0.0944	0.7676
X_3_: Sonication Time	42,077.55	1	42,077.55	2.69	0.1451
X_1_ X_2_	49,379.51	1	49,379.51	3.15	0.1190
X_1_ X_3_	1.593 × 10^5^	1	1.593 × 10^5^	10.18	0.0153
X_2_ X_3_	984.39	1	984.39	0.0629	0.8092
X_1_^2^	5.705 × 10^5^	1	5.705 × 10^5^	36.45	0.0005
X_2_^2^	44,571.95	1	44,571.95	2.85	0.1354
X_3_^2^	7298.83	1	7298.83	0.4663	0.5167
**Residual**	1.096 × 10^5^	7	15,653.00		
Lack of Fit	47,862.99	3	15,954.33	1.03	0.4674
Pure Error	61,707.98	4	15,427.00		
**Cor Total**	3.207 × 10^6^	16			

**Table 5:** ANOVA results for the zeta potential of CTX–APA nanoconjugates.

Source	Sum of Squares	Degrees of Freedom	Mean Square	F-Value	*p*-Value
Model	109.99	3	36.66	58.12	<0.0001
X_1_: CTX Concentration	109.08	1	109.08	172.89	<0.0001
X_2_: Incubation Time	0.2628	1	0.2628	0.4166	0.5299
X_3_: Sonication Time	0.6555	1	0.6555	1.04	0.3266
Residual	8.20	13	0.6309		
Lack of Fit	7.33	9	0.8139	3.71	0.1095
Pure Error	0.8765	4	0.2191		
Cor Total	118.20	16			

**Table 6:** Optimized independent variable levels and the predicted and observed responses of the optimized CTX–APA nanoconjugate.

Variables	X_1:_ CTX Concentration (mM)	X_2_: Incubation Time (min)	X_3_: Sonication Time (min)
Optimal values	1.00	66.40	10.0
	Predicted value	Observed value	Error %
Vesicle size (nm)	52.57	60.27 ± 2.8	12.78
Zeta potential (mV)	25.12	23.67± 2.66	6.13

**Table 7:** Histological features of wound healing in diabetic rats treated topically with CTX–APA on day 10.

	GT	RE	CD	IC	Phase I	Phase II	Phase III
Untreated Control	+	−	+	+++	++	++	−
Hydrogel	++	−	+	++	++	++	−
CTX	++	+	+	+	+	++	++
APA	+	+	++	−	+	++	++
CTX–APA	++	++	+++	+/−	−	+++	+++
Positive Control	++	+	++	+	+	+++	++

GT = granulation tissue; RE = re-epithelization; CD = collagen deposition; IC = inflammatory cell infiltration; Phase I = inflammation phase; Phase II = proliferation phase; Phase III = re-modeling phase; (−): absent; (+): mild; (++): moderate; (+++) severe.

**Table 8:** Effect of optimized CTX–APA nanoformula on oxidative stress in wounded skin of diabetic rats.

	SOD (Unit/mg Protein)	CAT (Unit/mg Protein)	MDA (nmol/mg Protein)	GSH (nmol/mg Protein)
Diabetic Untreated	5.22 ± 0.63	0.58 ± 0.07	8.60 ± 0.97	1.43 ± 0.15
Hydrogel	5.48 ± 0.66	0.61 ± 0.07	8.20 ± 0.92	1.70 ± 0.21
CTX	6.85 ^a^ ± 0.77	1.13 ^a^ ± 0.12	6.70 ^b^ ± 0.72	2.66 ^a,b^ ± 0.28
APA	7.41 ^a,b^ ± 0.86	1.24 ^a^ ± 0.14	7.21 ^a,b^ ± 0.80	2.23 ^a,b^ ± 0.24
CTX–APA	9.32 ^a,b,c,d^ ± 1.10	2.75 ^a,b,c,d^ ± 0.29	4.31 ^a,b,c,d^ ± 0.57	3.10 ^a,b,c,d^ ± 0.32
Positive Control	8.83 ^a,b,c^ ± 0.95	2.28 ^a,b,c,d^ ± 0.23	5.44 ^a,b,c,d^ ± 0.67	2.70 ^a,b,e^ ± 0.30

Data are displayed as means ± SD (*n* = 6). One-way ANOVA followed by Tukey’s test was performed and the significance level was set to *p* < 0.05 for all analyses. ^a^ Significant difference from diabetic untreated group. ^b^ Significant difference from hydrogel. ^c^ Significant difference from CTX. ^d^ Significant difference from APA. ^e^ Significant difference from CTX–APA.

## Data Availability

All data generated or analyzed in the current study are included in the published article.
